# High-Dose Selenium Induces Ferroptotic Cell Death in Ovarian Cancer

**DOI:** 10.3390/ijms24031918

**Published:** 2023-01-18

**Authors:** Jung-A Choi, Elizabeth Hyeji Lee, Hanbyoul Cho, Jae-Hoon Kim

**Affiliations:** Department of Obstetrics and Gynecology, Gangnam Severance Hospital, Yonsei University College of Medicine, Seoul 03722, Republic of Korea

**Keywords:** ovarian cancer, selenium, ferroptosis, lipid peroxidation, GPx4

## Abstract

Selenium is a promising multi-target chemotherapeutic agent with controversial clinical results. Hence, reassessing the anticancer effects of Se is necessary to clearly understand the potential of high-dose selenium in cancer treatment. Here, we observed that high-dose sodium selenite (SS) significantly decreased the proliferation and increased the death of ovarian cancer cells, mediated by an increased generation of reactive oxygen species. Notably, high-dose SS decreased the levels of glutathione peroxidase (GPx), a selenoprotein with antioxidant properties, without altering other selenoproteins. Furthermore, high-dose SS triggered lipid peroxidation and ferroptosis, a type of iron-dependent cell death, due to dysregulated GPx4 pathways. We demonstrated that intravenous high-dose SS significantly reduced the tumor growth and weight in SKOV3-bearing mice. Consistent with our in vitro results, mice with SKOV3 cells treated with high-dose SS showed decreased GPx4 expression in tumors. Therefore, we highlight the significance of high-dose SS as a potential chemotherapeutic agent for ovarian cancer. High-dose SS-mediated ferroptotic therapy integrating glutathione depletion and ROS generation is a promising strategy for cancer therapy.

## 1. Introduction

Epithelial ovarian cancer is one of three gynecological cancers with the highest prevalence and mortality rates [1], with more than two-thirds of patients with ovarian cancer diagnosed at an advanced stage due to the absence of effective, early diagnostic markers. Moreover, 85% of patients with primary advanced epithelial ovarian cancer develop cancer recurrence and anticancer drug resistance, requiring the urgent development of new drugs and therapeutic strategies to improve survival and enhance the chemotherapy response rate.

Ferroptosis, discovered in 2012, is a new type of iron-dependent cell death that differs from apoptosis, necrosis, and autophagy [2]. It is characterized by the accumulation of iron and lipid peroxides during cell death [3,4]. Cell morphology in ferroptosis differs from that during necrosis, apoptosis, and autophagy [5]. Ferroptosis plays an essential regulatory role in the occurrence and development of several diseases; recent research has focused on its role in the treatment and prognosis improvement of related diseases in multiple systems, such as neurodegenerative diseases, acute kidney injury, hepatic disease, gastrointestinal and pancreatic diseases, and cancer [6]. Ferroptosis-inducing factors can directly or indirectly affect the glutathione peroxidase (GPx) system through distinct mechanisms, resulting in reduced antioxidant activity and accumulation of lipid peroxidation products in cells, ultimately leading to oxidative cell death [6,7]. Of the GPx family members, GPx4 is critically involved in ferroptosis regulation by inhibiting lipid peroxide formation [8]. GPx4 detoxifies lipid peroxides produced by oxidative stress by converting glutathione (GSH) to oxidative glutathione and reducing cytotoxic lipid peroxides to alcohol [8]. Loss of GPx4 activity leads to lipid peroxide accumulation, which is a marker of ferroptosis [8]. However, ferroptosis is a relatively recent discovery, and several limitations remain in the development and use of any single ferroptosis-related drug as a therapeutic agent for intractable diseases.

Selenium (Se) is an essential mineral, with its deficiency linked to an increased risk of developing diseases, such as cardiovascular disorders, diabetes, and cancer [9,10]. It primarily functions in the antioxidant pathway via selenoproteins that contain selenium, including GPx, selenoprotein P (SELENOP), and thioredoxin reductases (TrxR) [11]. Se can act both as an oxidant and antioxidant in a metabolite- and dose-dependent manner [12]. At low doses, Se exhibits antioxidant properties and protects cells against oxidative damage, while high Se levels cause cancer cell death by increasing reactive oxygen species (ROS)-induced DNA damage [13,14]. Furthermore, Se compounds can induce tumor cell apoptosis through different pathways according to the cell type and compound structure [15]. These anticancer properties of Se have led to its extensive clinical evaluation as a chemotherapeutic agent. However, clinical studies have shown variable and conflicting results depending on the type and dose of selenium used. Therefore, the efficacy of selenium as a potential anticancer drug needs to be re-evaluated based on tumor characteristics, types, and optimal Se dosage.

Here, we aimed to investigate the efficacy of high-dose sodium selenite (SS) as an anticancer agent in ovarian cancer, and validate the anticancer-effective dose range of intravenously administered high-dose SS. In addition, we evaluated its ability to induce ferroptosis in intractable ovarian cancer.

## 2. Results

### 2.1. High-Dose SS Induces Cell Death in Ovarian Cancer Cells

**Figure 1:**
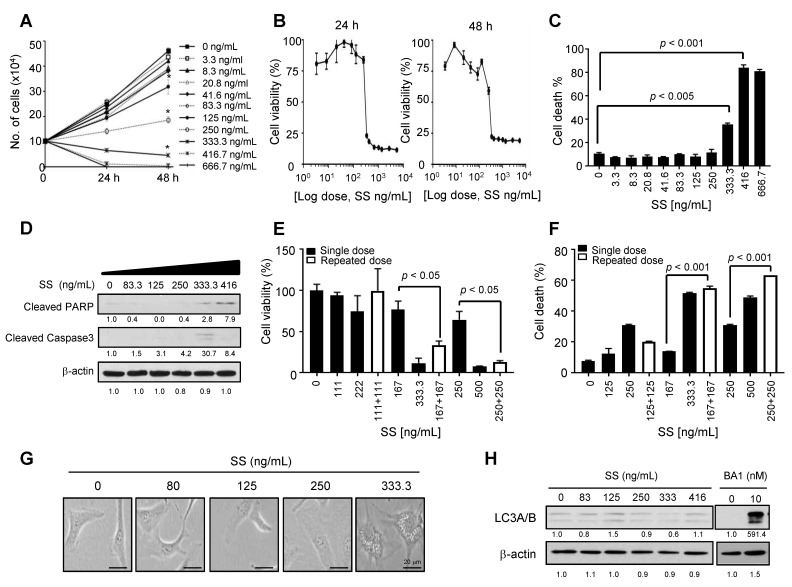
High-dose sodium selenite (SS) induces cell death in ovarian cancer cells. (**A**) Cell proliferation assay after SS treatment. SKOV3 cells were treated with SS at the indicated concentrations for 24 or 48 h. High-dose SS strongly inhibited cell proliferation. * *p* < 0.05. (**B**) Cell viability was determined by MTT assay after SS treatment for 24 or 48 h. (**C**) SKOV3 cells with SS exposure for 24 h were stained with a Annexin V-FITC/PI apoptosis detection kit. Annexin V-FITC staining and PI incorporation were measured in cells with a FACSCalibur flow cytometer and analyzed using FlowJo_v10.8.1 software. Cell death populations (%) correspond to the Annexin V-positive plus PI-positive population. (**D**) Cell lysates were subjected to immunoblotting with an anti-PARP antibody and anti–caspase-3 antibody. Anti-β-actin was used as the loading control. The numbers below each blot represent densitometric values. (**E**,**F**) Repeated SS administration exhibits a similar cell death effect as single administration. SKOV3 cells were treated with a single or repeated dose of SS. Repeated administration was performed twice at 24 h intervals with a halved dose as the single administration dose. Cell viability and cell death were determined by the MTT assay (**E**) and Annexin V assay (**F**), respectively. [Single dose: 125 ng/mL, 167 ng/mL, 222 ng/mL, 250 ng/mL, 333.3 ng/mL, and 500 ng/mL; repeat dose: 111 + 111 ng/mL, 125 + 125 ng/mL, 167 + 167 ng/mL, and 250 + 250 ng/mL]. (**G**) Differential morphological changes after high-dose SS treatments. SKOV3 cells were treated with SS (80, 125, 250, and 333.3 ng/mL). After 24 h, the cells were visualized using bright field microscopy. SS induced the formation of cytoplasmic vacuoles. Arrows indicate enlarged vacuoles. (**H**) Immunoblotting of LC3A/B in SS-treated SKOV3 cells. Bafilomycin A1 (BA1, 10 nM) was used as the positive control for detecting LC3B accumulation. Cell lysates were subjected to immunoblotting with an anti-LC3A/B antibody. Anti-β-actin was used as the loading control. The numbers below each blot represent densitometric values.

We evaluated the effect of SS on cell proliferation, viability, and cell death using the ovarian cancer cell line SKOV3 to investigate the anticancer effects of high-dose SS in ovarian cancer. SS significantly inhibited the cell proliferation of SKOV3 cells in a dose-dependent manner (Figure 1A). The 3-(4,5-dimethylthiazol-2-yl)-2,5-diphenyltetrazolium bromide (MTT) assay revealed that SS concentrations > 250 ng/mL significantly decreased the viability of ovarian cancer cells after 24 and 48 h (Figure 1B), with a half maximal inhibitory concentration (IC_50_) of 313 ng/mL for cell viability after 24 h. Moreover, SS concentrations higher than 333.3 ng/mL induced significant cell death, as measured by the annexin V assay, with weakly induced cell death at SS concentrations below 250 ng/mL (Figure 1C and Figure S1A). Thus, we selected SS at 333.3 ng/mL as the effective dose for cell death. Next, we investigated whether the observed cell death was apoptotic by assaying the levels of cleaved forms of PARP and caspase-3 and observed weak apoptosis after SS treatment (Figure 1D). Furthermore, we determined whether the anticancer effects of high-dose SS were mediated acutely by comparing the effects of repeated vs. single administration of SS. We observed similar cell viability (Figure 1E) and cell death (Figure 1F and Figure S1B) after two SS administrations of 167 ng/mL at 24 h intervals compared with those after a single SS administration of 333.3 ng/mL, suggesting that the anticancer effect of high-dose SS does not occur acutely due to high drug concentrations. These results indicate that high-dose SS exhibits potent anticancer effects against ovarian cancer.

### 2.2. High-Dose SS Induces Large Cytoplasmic Vacuoles in Ovarian Cancer Cells

We observed that high-dose SS (≥333.3 ng/mL) significantly induced the formation of cytoplasmic vacuoles compared to SS doses below 125 ng/mL (Figure 1G), with similar effects seen after repeated and single drug administrations (Figure S2). Interestingly, cytoplasmic vacuoles were not generated after treatment with chemotherapeutic agents, such as carboplatin and paclitaxel, despite cell death induction (Figures S3 and S4), suggesting that high-dose SS induces the formation of large cytoplasmic vacuoles in ovarian cancer cells, unlike other anticancer drugs.

The accumulation of autophagic vacuoles is a characteristic morphological feature of autophagy-mediated cell death [16]. It is well documented that microtubule-associated protein 1 light chain 3 (LC3) plays a critical role in the formation of cytoplasmic vacuoles that mediate autophagic cell death. Thus, using western blotting, we measured the levels of LC3B, a universal autophagy marker, after SS administration. Bafilomycin A1, which causes LC3B accumulation, was used as positive control. However, we did not observe altered LC3B expression levels (Figure 1H), suggesting that SS-mediated cell death is not correlated with autophagic cell death.

### 2.3. High-Dose SS Induces ROS Generation and Reduces GPx4 Expression in Ovarian Cancer

**Figure 2:**
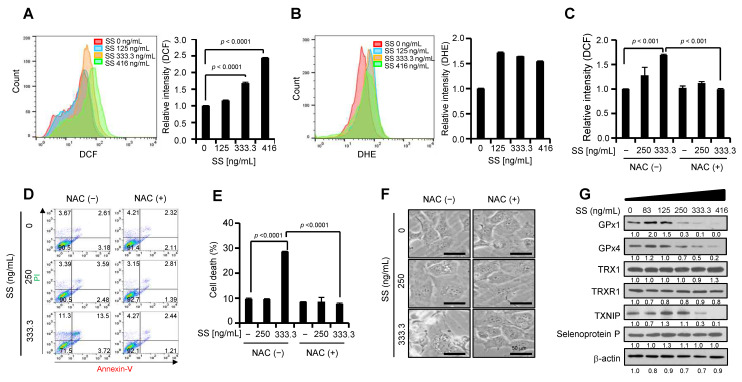
Effect of high-dose SS on the redox system in ovarian cancer cells. (**A**,**B**) Generation of intracellular ROS after SS treatment. SKOV3 cells were treated with SS at the indicated doses. After 24 h, the cells were incubated with 5 μM of H_2_DCFDA (**A**) or 2 μM of DHE (**B**) for 15 min at 37 °C. The cells were analyzed by flow cytometry. Relative fluorescence intensities were calculated using FlowJo_v10.8.1 software. (**C**) Impaired ROS generation by NAC in SS-treated SKOV3 cells. SKOV3 cells were pretreated with 5 mM NAC for 30 min and then treated with SS (250 ng/mL and 333.3 ng/mL) for 24 h. Intracellular ROS generation was measured by flow cytometry after H_2_DCFDA staining. (**D**,**E**) Prevention of cell death by ROS scavenger in SS-induced cell death. SKOV3 cells were pretreated with 5 mM NAC for 30 min and then treated with SS (250 ng/mL and 333.3 ng/mL) for 24 h. Cell death was estimated with a FACSCalibur flow cytometer using an Annexin V/PI apoptosis detection kit (**D**). Cell death populations (%) correspond to the Annexin V-positive plus PI-positive populations (**E**). (**F**) Reversed morphological changes by NAC on high-dose SS-mediated cytoplasmic vacuole formation. SKOV3 cells were pretreated with 5 mM NAC for 30 min and then treated with SS (250 ng/mL and 333.3 ng/mL) for 24 h. Then, the cells were visualized using bright field microscopy. (**G**) Effect of SS on GPx4 expression. SKOV3 cells were incubated with SS at concentrations of 0–416 ng/mL. After 24 h, the cells were harvested and subjected to immunoblotting with the indicated antibodies. Anti-β-actin was used as the loading control. The numbers below each blot represent densitometric values.

The cytotoxic effects of selenium drive apoptosis by redox cycling [17]. Thus, we investigated whether high-dose SS is involved in ROS generation. SS-treated SKOV3 cells were stained with 2′,7′-dichlorodihydrofluorescein diacetate (H_2_DCFDA), a fluorogenic dye that measures hydrogen peroxide, hydroxyl, peroxyl, and ROS activity within cells [18]. We observed increased intracellular ROS levels in an SS dose-dependent manner (Figure 2A) and obtained similar results after staining with dihydroethidium (DHE), a superoxide indicator (Figure 2B). Moreover, treatment with N-acetyl cysteine (NAC), an antioxidant and GSH precursor, prevented high-dose SS-mediated ROS generation and cell death (Figure 2C–F), as well as cytoplasmic vacuoles, suggesting that high-dose SS causes cell death by excessive ROS production in ovarian cancer cells. Next, we investigated the role of SS-mediated ROS generation in selenoprotein homeostasis by examining the effect of SS administration on the expression of selenoproteins and their related proteins (Figure 2G). We found that a high dose of SS reduced the expression of GPx1, GPx4, and TXNIP, whereas the expression of TRX1, TRXR1, and selenoprotein P was unaltered, indicating that a high dose of SS inhibits GPx1 and GPx4 expression. These results suggest that high-dose SS dysregulates the redox state, resulting in GPx1/4 deficiency and ROS accumulation. 

### 2.4. Selenium Induces Lipid Peroxidation and Ferroptosis-Mediated Cell Death in Ovarian Cancer Cells

**Figure 3:**
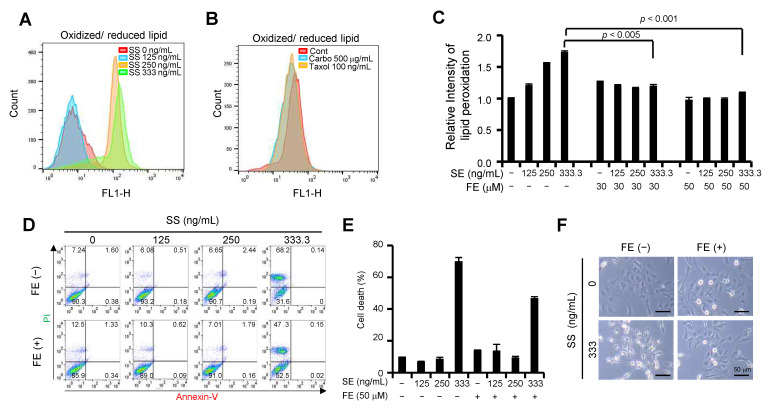
High-dose SS triggers lipid peroxidation in ovarian cancer cells. (**A**) Increased lipid peroxidation induced by high-dose SS in SKOV3 cells. SS-treated SKOV3 cells for 24 h were incubated with C11-BODIPY 581/591, a lipid peroxidation sensor, for 15 min and analyzed using flow cytometry. Oxidized lipids were analyzed using FlowJo_v10.8.1 software. (**B**) Effect of carboplatin or paclitaxel on lipid peroxidation in SKOV3 cells. SKOV3 cells treated with the drug for 24 h were incubated with C11-BODIPY 581/591 for 15 min and analyzed using flow cytometry. Oxidized lipids were analyzed using FlowJo_v10.8.1 software. (**C**) Impaired lipid peroxidation by a ferroptosis inhibitor in SKOV3 cells with high-dose SS. Cells were pretreated with ferrostatin-1 at a concentration of 30 μM or 50 μM for 30 min and then incubated with SS. After 24 h, the cells were incubated with C11-BODIPY 581/591 for 15 min and analyzed by flow cytometry. Relative intensities were calculated using FlowJo_v10.8.1 software and presented as lipid peroxidation. (**D**,**E**) Prevention of cell death by a ferroptosis inhibitor in high-dose SS-exposed SKOV3 cells. The cells were pretreated with ferrostatin-1 at a concentration of 50 μM for 30 min and then incubated with SS. After 24 h, cell death was determined by a Annexin V-FITC/PI apoptosis detection kit. Annexin V-FITC staining and PI incorporation were measured in cells with a FACSCalibur flow cytometer (**D**) and analyzed with FlowJo_v10.8.1 software. Cell death populations (%) correspond to the Annexin V-positive plus PI-positive populations (**E**). (**F**) Reversed morphological changes by the ferroptosis inhibitor in SS-exposed SKOV3 cells. Reversed morphological shapes were visualized using bright field microscopy.

Hydrogen peroxide is converted to hydroxyl radicals (OH^−^) through the Fenton reaction or H_2_O via the GPx system [19]. Hydroxyl radicals are responsible for membrane lipid peroxidation as well as DNA and protein damage [20]. Therefore, we investigated whether high-dose SS is involved in lipid peroxidation. Cells treated with varying SS concentrations were stained with C11-BODIPY (581/591), a fluorescent radioprobe used to detect lipid peroxidation. The lipid peroxidation assay revealed that high-dose SS significantly enhanced lipid peroxidation compared to lower SS doses (Figure 3A). Interestingly, carboplatin or paclitaxel treatment did not affect lipid peroxidation (Figure 3B). Next, we investigated whether a high dose of SS is involved in ferroptotic cell death due to lipid peroxidation, and whether GPx4 deficiency is implicated in ferroptosis. Notably, treatment with ferrostatin (30 μM and 50 μM), a ferroptosis inhibitor, prevented high-dose SS-induced lipid peroxidation (Figure 3C). Moreover, ferrostatin treatment also prevented high-dose SS-induced cell death (Figure 3D–E). Similarly, cytoplasmic vacuolation induced by high-dose SS was significantly inhibited by ferrostatin treatment (Figure 3F). These results suggest that high-dose SS induces ferroptosis in ovarian cancer.

### 2.5. Anticancer Effect of High-Dose SS in SKOV3 Cell-Induced Ovarian Cancer Model

**Figure 4:**
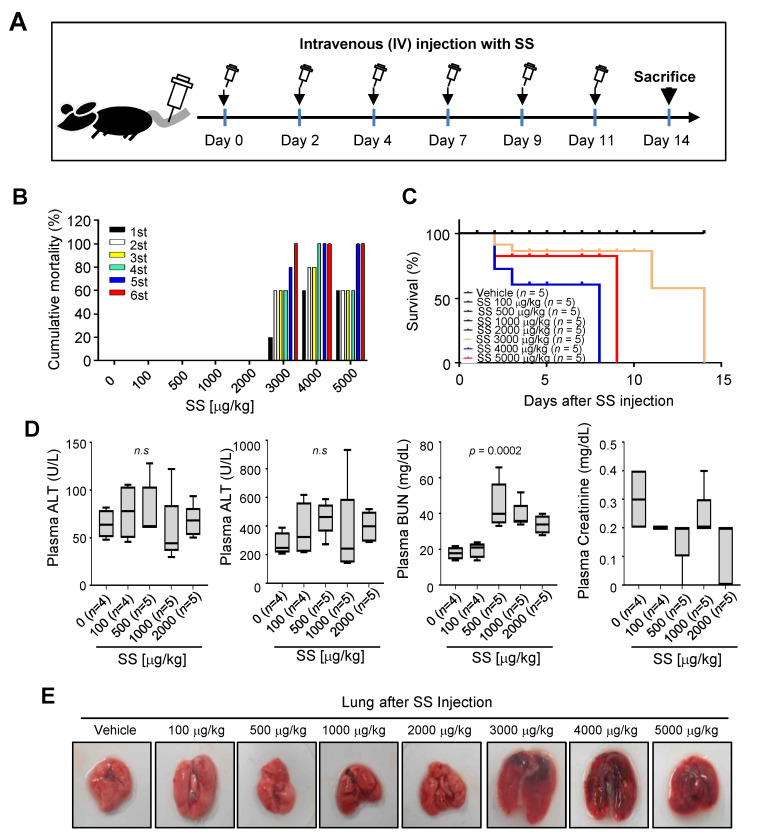
Lethal dose screening of high-dose SS in BALB/c nude mice. (**A**) Schematic description of the experimental design. SS (0–5000 μg/kg) was injected thrice weekly into BALB/c nude mice intravenously through the tail vein at the indicated time points for 2 weeks. Mortality and mouse behavior was monitored thrice weekly. Mice were euthanized after 2 weeks. (**B**,**C**) Animal mortality and survival curves following the intravenous administration of SS and vehicle in BALB/c mice. (**B**) The mortality rate is the cumulative mortality rate per SS injection. Note that mice died at SS concentrations above 3000 μg/kg. (**C**) Kaplan–Meier survival curve shows normal survival rates at SS concentrations below 2000 μg/kg. (**D**) Effect of SS on liver and kidney toxicity in the plasma of BALB/c nude mice. Alanine aminotransferase (ALT), aspartate aminotransferase (AST), blood urea nitrogen (BUN), and creatinine levels. (**E**) Representative pathological analysis in the lungs of SS-injected BALB/c nude mice. *n* = number of mice.

We investigated the effect of a lethal dose of SS in BALB/c nude mice to evaluate its clinical applicability. BALB/c nude mice were intravenously injected with 100–5000 μg/kg doses of SS three times a week for 2 weeks (Figure 4A). Cumulative mortality (%) analysis revealed that the groups treated with SS concentrations between 100 and 2000 μg/kg survived, but the groups treated with SS concentrations above 3000 μg/kg died (Figure 4B). Kaplan–Meier curves revealed no significant difference in survival periods below SS concentrations of 2000 μg/kg, and significantly shortened survival periods at SS doses above 3000 μg/kg (Figure 4C). Next, we analyzed the plasma levels of aspartate aminotransferase (AST), alanine aminotransferase (ALT), blood urea nitrogen (BUN), and creatinine in BALB/c nude mice after SS treatment to determine the effect of high-dose SS on hepatotoxicity and nephrotoxicity (Figure 4D). SS doses ranging from 100 to 2000 μg/kg did not affect ALT or creatinine levels. BUN levels were slightly elevated at SS doses above 500 μg/kg (Figure 4D). Pulmonary edema was observed at SS doses of 3000 μg/kg or above (Figure 4E).

**Figure 5:**
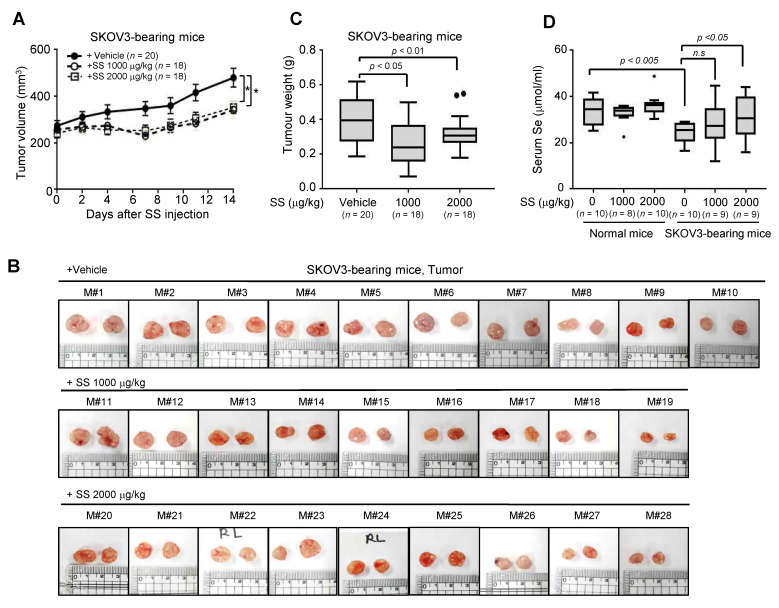
Anticancer effect of high-dose SS in SKOV3-bearing mice xenograft mouse models. (**A**–**C**) SKOV3-bearing BALB/c nude mice were administered SS at 1000 μg/kg and 2000 μg/kg or vehicle thrice weekly for 2 weeks. Animals representing the six treatment groups are shown. Tumor size (mm^3^) was monitored thrice weekly (**A**). Mice were euthanized after 2 weeks. The tumor was isolated (**B**) and its weight (g) was measured (**C**). *n* = number of tumor. (**D**) Levels of selenium in SKOV3-bearing BALB/c nude mice with SS. Serum selenium levels was determined using ELISA assay (**D**). *n* = number of mice.

Based on these observations, we analyzed the preclinical anticancer effect of high-dose SS in the SKOV3 cell-induced ovarian cancer model induced by intravenous SS injections thrice weekly for 2 weeks at 1000 and 2000 μg/kg. As shown in Figure 5A, both SS doses significantly reduced tumor growth rate (*p* < 0.05) and decreased tumor weight compared to those in the control group (Figure 5B,C; control vs. 1000 μg/kg SS vs. 2000 μg/kg SS, 0.390 ± 0.028 g vs. 0.268 ± 0.030 g vs. 0.327 ± 0.022 g, respectively). Importantly, SKOV3-bearing BALB/c mice showed selenium deficiency compared to non-SKOV3-bearing mice (Figure 5D; control, 34.0 ± 1.9 Umol/mL; SKOV3-bearing, 24.3 ± 1.5 Umol/mL; *p* < 0.005). These phenomena were reversed by administering SS (Figure 5D), suggesting that high-dose selenium has potential as an anticancer drug for ovarian cancer. 

### 2.6. High-Dose SS Inhibits GPx4 Expression in Tumor Tissues of SKOV3-Bearing Ovarian Cancer Model Mice

**Figure 6:**
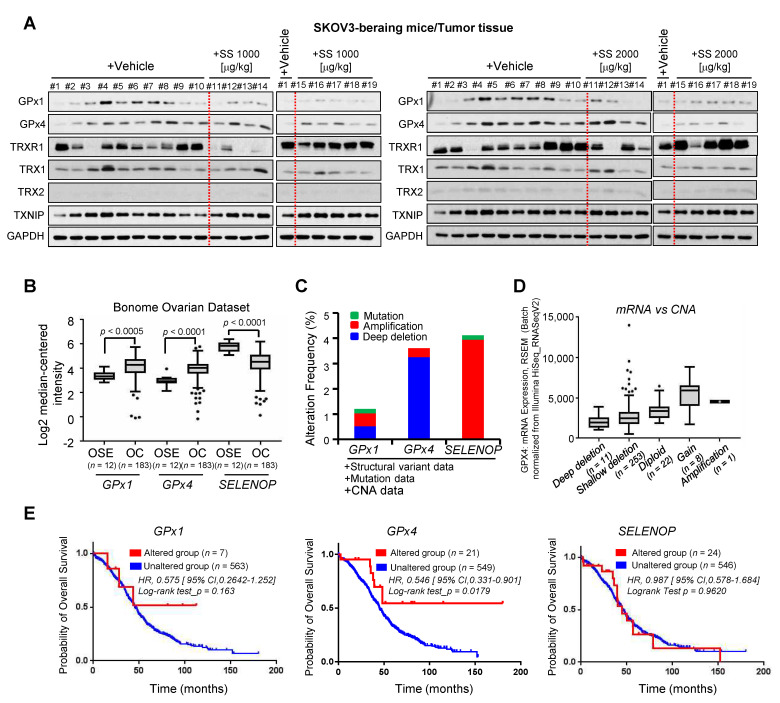
Effect of SS on GPX expression in SKOV3-bearing mice. (**A**) Expression of selenoproteins in tumors of SKOV3-bearing mice. Tumor tissue samples were harvested from mice and lysed for western blot analysis. Lysates were immunoblotted with GPx1, GPx4, TXNIP, TRX1, or selenoprotein P antibodies. GAPDH was used as the loading control. (**B**) mRNA levels of *GPX1*, *GPX4,* and *SELENOP* in patients with ovarian cancer obtained from the Oncomine public database. (**C**) Alterations in the frequencies of *GPx1, GPx4*, and *SELENOP* genes in ovarian cancer were assessed using the cBioPortal for Cancer Genomics. (**D**) *GPX4* mutations in ovarian cancer led to deep deletions. Plot shows GPx4 mRNA expression versus copy number alteration due to different mutations resulting in deep deletions, shallow deletions, or diploid or gain of functions. (**E**) Kaplan–Meier plots comparing overall survival (OS) in patients with ovarian cancer with or without genetic alterations in *GPX1, GPX4,* and *SELENOP* using cBioPortal.

Here, we showed that high-dose SS inhibited the expression of GPx4, a ferroptosis inhibitor, in ovarian cancer cells in vitro. We analyzed the expression of GPx4 and selenium-related proteins in an SS-administered mouse model. Immunoblotting results revealed significantly reduced GPx4 levels in the tumor tissues of SKOV3-bearing mice models compared to those in vehicle-treated groups (Figure 6A and Figure S5), with no change in the levels of other selenium proteins. This suggests that high-dose SS causes GPx4 deficiency in SKOV3-bearing mouse models.

Lastly, we monitored the expression of GPX1, GPX4, and SELENOP genes in patients with ovarian cancer using the publicly available Oncomine database to elucidate the clinical significance of GPx4 in ovarian cancer. Oncomine revealed that the mRNA levels of GPx1 and GPx4 were strongly elevated in ovarian cancer tissues compared to those in normal ovarian tissues, whereas SELENOP levels were decreased in ovarian cancer tissues (Figure 6B). Next, we investigated the relevance of GPx4 through integrative analysis of complex cancer genomics and clinical profiles using cBioPortal. We observed an increase in the alteration frequency of these genes (mutation, amplification, and deep deletion) in ovarian cancer tissues (Figure 6C). Among them, the alteration frequencies of amplification and deep deletion in GPX4 were 0.34% (2/584) and 3.25% (19/584), respectively, suggesting that most alterations in GPX4 were of the deep deletion type (Figure 6C). Copy number alteration analysis revealed impaired GPX4 mRNA expression in the deep deletion type (Figure 6D), suggesting that deep deletion of GPX4 may lead to decreased mRNA expression. Notably, Kaplan–Meier plots showed that the group with altered GPX4 genes displayed longer overall survival (*p* < 0.05) compared to the group with unaltered GPX4 genes (Figure 6E), whereas alterations of GPX1 and SELENOP were not significantly associated with overall survival.

## 3. Discussion

In this study, we established the clinical applicability of high-dose SS by evaluating its anticancer effect after intravenous administration in ovarian cancer xenograft mouse models. In addition, we found that the anticancer effect of high-dose SS was due to increased production of hydroxyl radicals, leading to ferroptosis-mediated cell death via lipid peroxidation, distinct from that induced by other anticancer drugs.

Se has been evaluated extensively as a promising chemotherapeutic agent with conflicting results depending on the type and dose of Se used. Selenium compounds can be categorized as organic forms with selenocysteine (SeCys), selenomethionine (SeMet), and Se-methylselenocysteine (MSC), as well as inorganic forms with selenate and selenite [21]. Maiko et al. reported that different selenium metabolites exerted variable effects on cell apoptosis, with SeMet increasing apoptosis more than MSC in p53-positive A549 cells, and selenite increasing apoptosis compared to SeMet and MSC in p53-mutated HSC-3 cells [22]. Jin et al. observed that SS inhibited growth in ovarian carcinoma cells in vitro, whereas an intraperitoneal SS injection (1.5 mg/kg) had no effect on tumor growth either alone or when combined with paclitaxel in ovarian cancer mouse models [23]. In the Nutritional Prevention of Cancer (NPC) Trial, supplementation with Se-enriched yeast reduced the incidence of several cancers, including lung, prostate, and colorectal cancers, while breast cancer, bladder cancer, and leukemia showed no reduction in occurrence [24]. These results suggest that the mechanisms of Se action may depend on Se type, dose, schedule, and tumor profile. Therefore, we evaluated the anticancer effect of intravenous administration of liquid SS in an ovarian cancer mouse model. We identified the effective dose (1000–2000 μg/kg) of SS for anticancer effects in an ovarian cancer mouse model through tail injection three times per week for 2 weeks. Thus, we propose that SS inhibits tumor growth when administered intravenously. To the best of our knowledge, this is the first report of the use of high-dose aqueous SS as a treatment for cancer through intravenous administration. 

Although Se is widely recognized as an antioxidant that reduces ROS levels, growing evidence shows that Se compounds exert both oxidant as well as antioxidant effects depending on their chemical form and concentration [25,26,27,28]. Se has been shown to exert cytotoxic effects on cancer cells by inducing oxidative stress after extensive ROS generation and DNA damage. Selenite-induced apoptosis results from superoxide-mediated and p53-dependent mitochondrial damage in prostate cancer [12]. SS inhibits the proliferation and metastasis of renal cell carcinoma through ROS-mediated nuclear factor-κB signaling [29]. SS induces apoptosis and autophagy in lung cancer A549 cells by increasing intracellular ROS levels [30]. Consistent with these reports, we observed that high-dose SS triggers ROS generation, and treatment with NAC, an antioxidant, inhibited high-dose SS-induced cell death in ovarian cancer cells, indicating that high-dose SS-induced cell death is mediated by ROS generation. Although selenoproteins are the predominant biologically functional form of Se synthesized by the Se metabolic system [31], we found that high doses of SS reduced GPx4 expression in an SS-dose-dependent manner, but did not affect the expression of other selenoproteins, such as selenoprotein P, TRXR1, and TRX1, in ovarian cancer cells in vitro and in vivo. The mechanism underlying GPx4 depletion by high-dose SS remains unclear; however, a possible mechanism could involve Wnt/β-catenin signaling. A recent study suggested that the beta-catenin/TCF4 transcription complex directly binds to the GPx4 promoter and regulates its expression to impair cellular lipid peroxidation, thus inhibiting ferroptosis [32]. These observations warrant further research on GPx4 mechanisms. 

Lipid oxidation and ferroptosis are regulated by multiple pathways. While several studies have shown the regulatory mechanisms of ferroptosis, including the Fenton reaction, ROS, the System-Xc−-GSH-GPX4 pathway, the ferroptosis suppressor protein 1 (FSP1)–ubiquinone system, and the p53-related pathway [3,4,5,19], a detailed understanding of its mechanisms remains unclear. The GPx4 system acts as a critical ferroptosis regulator by catalyzing the reduction of lipid peroxides via redox homeostasis [33,34,35]. GPx4 overexpression and knockdown has been shown to modulate the lethality of 12 ferroptosis inducers [35]. RSL-3, a pharmacological ferroptosis inducer, triggered colorectal cancer cell death via direct binding and inactivation of GPx4, resulting in the disruption of redox balance [34]. GPx4-mediated ferroptosis is promising for gefitinib resistance in triple-negative breast cancer [36]. Furthermore, GSH depletion has been linked to reduced GPx4 activity and ferroptosis in cancer cells [34,37,38]. Deletion of SLC7A11, a cystine-glutamine anti-transporter, decreased cystine import, downregulated GSH activity, and induced tumor ferroptosis [38]. Therefore, the GPx4 system is a key modulator of ferroptosis-mediated cell death. Interestingly, our public data analysis revealed that GPx4 is overexpressed in the ovaries of patients with ovarian cancer compared with that in healthy women with epithelial ovaries, and patients with altered GPx4 levels displayed a longer overall survival than the unaltered groups, suggesting that GPx4 status can play an important role in ovarian cancer progression. A recent study supports our hypothesis by suggesting that ferroptosis signatures (e.g., SLC7A111, GPx4, ALOX5, P53, and RPL8) are enriched in platinum-tolerant ovarian cancer cells harboring stemness feature, and GPx4 is significantly upregulated in platinum-tolerant cells, xenografts, patient-derived xenografts, and high grade serous ovarian cancer specimens after neoadjuvant chemotherapy [39]. Notably, we found that high-dose SS induced lipid peroxidation, ferroptosis, and GPx4 deficiency in ovarian cancer cells. NAC, a GSH precursor, inhibited high-dose SS-mediated ferroptosis and lipid peroxidation, indicating that high-dose SS triggers ferroptosis through an imbalanced redox status. Consistent with our in vitro results, the SKOV3-bearing mouse group with high-dose SS administration showed GPx4 deficiency and reduced tumor growth compared with the vehicle-treated ovarian cancer mouse groups. Thus, we postulated that, unlike other chemotherapy drugs, high-dose SS induces ferroptosis-mediated cell death through an abnormal GPx4-GSH-lipid peroxidation mechanism, resulting in anticancer effects. We believe that this discriminatory toxic pathway of SS can prevent the development of drug resistance in cancer cells by targeting multiple pathways during chemotherapy, and this complexity in the manifestation of toxic effects is of great pharmacological advantage.

Although the anticancer effect of high-dose SS was confirmed in in vivo and in vitro studies, and an appropriate dose was identified, there are a few limitations of this study. High doses of SS exerted a weak effect on the death of immortalized human ovarian surface epithelial cells at IC_50_ (Figure S6), and ferroptosis inhibitor treatment has a slight effect on this cell death. These observations suggest that high-dose SS induces cell death more sensitively in ovarian cancer cells than in the normal cells, implying that high-dose SS can also affect normal cells. However, repeated intravenous administration of SS, which has anti-cancer properties, did not show hepatotoxicity and pulmonary congestion symptoms, and did not induce mouse mortality. These in vivo results suggest that the anticancer effect of high-dose SS upon intravenous administration has a tumor-specific effect. Therefore, we propose that the anticancer effect of high-dose SS can be highly effective on tumor cells.

In summary, we have highlighted the significance of high-dose SS as a potential chemotherapeutic agent against ovarian cancer in vivo and in vitro. We suggest that the anticancer effect of high-dose SS is due to an unbalanced redox system caused by GPx4 deficiency, leading to ferroptosis-mediated cell death, which is distinct from other anticancer drugs. Currently, only a few basic studies have evaluated ferroptosis for clinical treatment of ovarian cancer. Therefore, high-dose SS-mediated ferroptotic therapy integrating GSH depletion and ROS generation is a promising cancer therapy, thereby aiding clinicians in monitoring cancer progress and providing optimal treatment options.

## 4. Materials and Methods

### 4.1. Cell Culture and Reagents

The human ovarian cancer cell line, SKOV3, was obtained from the American Type Culture Collection (Manassas, VA, USA). The cells were cultured in Dulbecco’s modified Eagle’s medium supplemented with 10% fetal bovine serum (HyClone, UT, USA) and 1% penicillin-streptomycin (Corning, Tewksbury, MA, USA). Ferrostatin-1 and NAC were purchased from Sigma-Aldrich (St. Louis, MO, USA). Carboplatin and paclitaxel were obtained from Boryung Pharmaceutical Co., Ltd. (Seoul, Republic of Korea) and BMS Pharmaceutical Korea (Seoul, Republic of Korea), respectively.

### 4.2. Cell Proliferation Assay

Cells were plated at a density of 1 × 10^5^ cells per well in 6-well plates. After 24 h, the cells were treated with SS and incubated at 37 °C for the indicated times (24, 48, and 72 h). Cell growth was measured by trypsinizing treated cells at each time point and counting them using the trypan blue exclusion method.

### 4.3. MTT Assay

Cell viability was measured using the MTT assay. Cells were briefly seeded onto 96-well plates at a density of 5 × 10^4^ cells/mL. After 24 h, cells were exposed to SS for 0–48 h at 37 °C, and subsequently incubated with the MTT solution for an additional 3 h at 37 °C. The remaining medium was disposed and dimethyl sulfoxide was added to each well to solubilize the precipitate. Optical density was measured at 570 nm using a VersaMax microplate reader (Molecular DEVICES Inc., San Jose, CA, USA), and cell viability was calculated. 

### 4.4. Cell Death Analysis

Cell death was determined using the eBioscience™ Annexin V-FITC Apoptosis Detection Kit (Invitrogen, CA, USA), according to the manufacturer’s instructions. Briefly, cells were treated with SS as described above before harvesting, and resuspended in binding buffer with 5 μL Annexin V-FITC and propidium iodide (PI) (20 μg/mL) at room temperature for 10 min. The cells were centrifuged for 5 min at 2600× *g* and the pellets were resuspended in a binding buffer. Finally, the percentage of cell death was evaluated by flow cytometry (Beckman Coulter Inc., Brea, CA, USA). The cells positive for Annexin V-FITC and PI were counted as dead cells.

### 4.5. Intracellular ROS Generation

To measure intracellular H_2_O_2_ and O^2−^, cells were incubated for 10 min with the cell-permeable probe, H_2_DCFDA (10 μM), an indicator of reactive oxygen diacetate, and the superoxide indicator DHE (10 μM). The cells were then resuspended in phosphate-buffered saline (PBS), and DCFDA and DHE fluorescence was measured using a FACSCalibur™ flow cytometer (Becton Dickinson, Franklin Lakes, NJ, USA). Values represent the mean ± standard deviation (SD) of DCFDA and DHE fluorescence. 

### 4.6. Lipid Peroxidation Assay

The C11 BODIPY 581/591 is a lipid-soluble ratiometric fluorescent indicator of lipid oxidation. The lipid peroxidation assay was performed using C11 BODIPY 581/591 (Thermo Fisher Scientific, Waltham, MA, USA), as described previously [40]. Cells were briefly plated at 4 ×10^4^ cells/well in 6-well plates and treated with SS at the indicated concentrations. After 24 h, the cells were treated with 2 µM C11 BODIPY 581/591 and analyzed using a FACSCalibur™ flow cytometer. 

### 4.7. Ovarian Cancer Xenograft Mouse Model

Four-week-old female BALB/c nude mice were purchased from Orient Bio (Seongnam, Republic of Korea) and housed in specific pathogen-free rooms. The mice were housed in a controlled temperature (25 °C) environment with a 12 h light/dark cycle and fed water and food (standard basal diet). All mice were allowed to acclimate for at least two weeks before the experiments. Sodium selenite pentahydrate (Ph. Eur.) was provided by Biosyn Arzneimittel GmbH (Fellbach, Germany). The study was reviewed and approved by the Institutional Animal Care and Use Committee of the Yonsei University Health System (IACUC Approval No. 2020-0229). The lethality of high-dose SS was evaluated by injecting BALB/c nude mice with SS (0, 100, 500, 1000, 2000, 3000, 4000, and 5000 μg/kg) three times per week at a volume of 200 μL for 2 weeks, and monitoring mice behavior and lethality. BALB/c nude mice were monitored for pulmonary edema and euthanized 2 weeks after the SS injection. Plasma levels of AST, ALT, BUN, and creatinine were evaluated at Chaon Ltd., Seoul, Republic of Korea.

The anticancer effects of sodium selenite on ovarian cancer were evaluated by subcutaneously implanting SKOV3 cells (2 × 10^6^ cells) in the left and right flanks of 5-week-old BALB/c nude mice. When the average size of the induced tumor was >250 mm^3^, SS was injected three times per week at a volume of 200 μL for 2 weeks. Tumor size was estimated by measuring the length and width of the tumor three times per week using a caliper. The mice were euthanized after 2 weeks and total body weight, tumor weight, tumor incidence, and number of tumor nodules were recorded at the time of euthanasia. The Selenium Assay Kit (Abbexa Ltd., Cambridge, UK) was used to measure serum selenium levels. The tumor volume was calculated using the formula V = (W^2^ × L)/2 and by using a caliper [41].

### 4.8. Western Blot Analysis

Cultured SKOV3 cells and mouse tumor tissues were lysed in ice-cold Cell Lysis Buffer (Cell Signaling Technology Inc., Beverly, MA, USA) containing a protease inhibitor. Cell lysates were resolved using 10% sodium dodecyl sulfate-polyacrylamide gel electrophoresis (SDS-PAGE) and transferred to a nitrocellulose membrane (Pall Corporation, Pensacola, IL, USA) using an electric transfer system. The membranes were incubated with the following antibodies: anti-GPx1 (Cat. #3286; 1:000), anti-GPx4 (Cat. #52455; 1:1000), anti-TRX1 (Cat. #2429; 1:1000), anti-TXNIP (Thioredoxin interacting protein) (Cat. #14715; 1:1000), (Cell Signaling Technology, Danvers, MA, USA), anti-selenoprotein P (Cat. #376585; 1:1000; Santa Cruz Biotechnology, Santa Cruz, CA, USA), and anti-β-actin (Sigma-Aldrich). The membranes were then incubated with horseradish peroxidase-conjugated goat anti-mouse or anti-rabbit secondary antibodies (Bio-Rad Laboratories, Hercules, CA, USA), and immunoreactive bands were visualized using enhanced chemiluminescence reagents (Santa Cruz Biotechnology, Santa Cruz, CA, USA). 

### 4.9. Public Databases

The mRNA levels of GPx1 and GPx4 in patients with ovarian cancer were determined by analysis of TCGA_Ovarian dataset, which is available through Oncomine. Alterations in the frequencies of GPx1, GPx4, and SELENOP genes in ovarian cancer were assessed by analysis of The Cancer Genome Atlas (TCGA) PanCancer Atlas Studies using the cBioPortal for Cancer Genomics.

### 4.10. Statistical Analysis

Results are expressed as the mean ± SD or standard error (SEM). Most statistical analyses were performed using Student’s *t*-test, Mann–Whitney test, or Spearman’s rank correlation coefficient using GraphPad Prism version 7 (GraphPad Software, Inc., La Jolla, CA, USA). Statistical significance was set at *p* < 0.05.

## Data Availability

Not applicable.

## Supplementary Materials

The following supporting information can be downloaded at: https://www.mdpi.com/article/10.3390/ijms24031918/s1.

## References

[1] Zeppernick F., Meinhold-Heerlein I., Meinhold-Heerlein Á.I. (2014). The new FIGO staging system for ovarian, fallopian tube, and primary peritoneal cancer. Arch. Gynecol. Obs..

[2] Hu Y.-J., Chen Y., Zhang Y.-Q., Zhou M.-Z., Song X.-M., Zhang B.-Z., Luo L., Xu P.-M., Zhao Y.-N., Zhao Y.-B. (1997). The protective role of selenium on the toxicity of cisplatin-contained chemotherapy regimen in cancer patients. Biol. Trace Element Res..

[3] Dixon Scott J., Lemberg K.M., Lamprecht M.R., Skouta R., Zaitsev E.M., Gleason C.E., Patel D.N., Bauer A.J., Cantley A.M., Yang W.S. (2012). Ferroptosis: An iron-dependent form of nonapoptotic cell death. Cell.

[4] Li J., Cao F., Yin H.L., Huang Z.J., Lin Z.T., Mao N., Sun B., Wang G. (2020). Ferroptosis: Past, present and future. Cell Death Dis..

[5] Yang W.S., Stockwell B.R. (2008). Synthetic lethal screening identifies compounds activating iron-dependent, nonapoptotic cell death in oncogenic-RAS-harboring cancer cells. Chem. Biol..

[6] Pan F., Lin X., Hao L., Wang T., Song H., Wang R. (2022). The Critical Role of Ferroptosis in Hepatocellular Carcinoma. Front. Cell Dev. Biol..

[7] Zhang G., Fang Y., Li X., Zhang Z. (2022). Ferroptosis: A novel therapeutic strategy and mechanism of action in glioma. Front. Oncol..

[8] Ursini F., Maiorino M. (2020). Lipid peroxidation and ferroptosis: The role of GSH and GPx4. Free Radic. Biol. Med..

[9] Rees K., Hartley L., Day C., Flowers N., Clarke A., Stranges S. (2013). Selenium supplementation for the primary prevention of cardiovascular disease. Cochrane Database Syst. Rev..

[10] Sanmartin C., Plano D., Font M., Palop J.A. (2011). Selenium and clinical trials: New therapeutic evidence for multiple diseases. Curr. Med. Chem..

[11] Sanmartín C., Plano D., Palop J.A. (2008). Selenium compounds and apoptotic modulation: A new perspective in cancer therapy. Mini-Rev. Med. Chem..

[12] Zhao R., Xiang N., Domann F.E., Zhong W. (2006). Expression of p53 enhances selenite-induced superoxide production and apoptosis in human prostate cancer cells. Cancer Res..

[13] Zeng H., Wu M., Botnen J.H. (2009). Methylselenol, a selenium metabolite, induces cell cycle arrest in G1 phase and apoptosis via the extracellular-regulated kinase 1/2 pathway and other cancer signaling genes. J. Nutr..

[14] Fang W., Han A., Bi X., Xiong B., Yang W. (2010). Tumor inhibition by sodium selenite is associated with activation of c-Jun NH_2_-terminal kinase 1 and suppression of beta-catenin signaling. Int. J. Cancer.

[15] Takahashi M., Sato T., Shinohara F., Echigo S., Rikiishi H. (2005). Possible role of glutathione in mitochondrial apoptosis of human oral squamous cell carcinoma caused by inorganic selenium compounds. Int. J. Oncol..

[16] González-Polo R.A., Boya P., Pauleau A.L., Jalil A., Larochette N., Souquère S., Eskelinen E.L., Pierron G., Saftig P., Kroemer G. (2005). The apoptosis/autophagy paradox: Autophagic vacuolization before apoptotic death. J Cell Sci..

[17] Brigelius-Flohé R., Flohé L. (2017). Selenium and redox signaling. Arch. Biochem. Biophys..

[18] Eruslanov E., Kusmartsev S. (2010). Identification of ROS using oxidized DCFDA and flow-cytometry. Methods Mol. Biol..

[19] Yagoda N., Von Rechenberg M., Zaganjor E., Bauer A.J., Yang W.S., Fridman D.J., Wolpaw A.J., Smukste I., Peltier J.M., Boniface J.J. (2007). RAS-RAF-MEK-dependent oxidative cell death involving voltage-dependent anion channels. Nature.

[20] Santos A.L., Preta G. (2018). Lipids in the cell: Organisation regulates function. Cell. Mol. Life Sci..

[21] Hariharan S., Dharmaraj S. (2020). Selenium and selenoproteins: It’s role in regulation of inflammation. Inflammopharmacology.

[22] Suzuki M., Endo M., Shinohara F., Echigo S., Rikiishi H. (2010). Differential apoptotic response of human cancer cells to organoselenium compounds. Cancer Chemother. Pharmacol..

[23] Park J.S., Ryu J.Y., Jeon H.-K., Cho Y.J., Park Y.A., Choi J.-J., Lee J.-W., Kim B.-G., Bae D.-S. (2012). The effects of selenium on tumor growth in epithelial ovarian carcinoma. J. Gynecol. Oncol..

[24] Clark L.C., Combs G.F., Turnbull B.W., Slate E.H., Chalker D.K., Chow J., Davis L.S., Glover R.A., Graham G.F., Gross E.G. (1996). Effects of selenium supplementation for cancer prevention in patients with carcinoma of the skin. A randomized controlled trial. Nutritional Prevention of Cancer Study Group. JAMA.

[25] Kuršvietienė L., Mongirdienė A., Bernatonienė J., Šulinskienė J., Stanevičienė I. (2020). Selenium Anticancer Properties and Impact on Cellular Redox Status. Antioxidants.

[26] Kim S.J., Choi M.C., Park J.M., Chung A.S. (2021). Antitumor Effects of Selenium. Int. J. Mol. Sci..

[27] Qi Y., Schoene N.W., Lartey F.M., Cheng W.H. (2010). Selenium compounds activate ATM-dependent DNA damage response via the mismatch repair protein hMLH1 in colorectal cancer cells. J. Biol. Chem..

[28] Zhou N., Xiao H., Li T.K., Nur-E-Kamal A., Liu L.F. (2003). DNA damage-mediated apoptosis induced by selenium compounds. J Biol. Chem..

[29] Liu X., Jiang M., Pang C., Wang J., Hu L. (2022). Sodium selenite inhibits proliferation and metastasis through ROS-mediated NF-κB signaling in renal cell carcinoma. BMC Cancer.

[30] Park S.-H., Kim J.-H., Chi G.Y., Kim G.-Y., Chang Y.-C., Moon S.-K., Nam S.-W., Kim W.-J., Yoo Y.H., Choi Y.H. (2012). Induction of apoptosis and autophagy by sodium selenite in A549 human lung carcinoma cells through generation of reactive oxygen species. Toxicol. Lett..

[31] Kang D., Lee J., Wu C., Guo X., Lee B.J., Chun J.S., Kim J.H. (2020). The role of selenium metabolism and selenoproteins in cartilage homeostasis and arthropathies. Exp. Mol. Med..

[32] Wang Y., Zheng L., Shang W., Yang Z., Li T., Liu F., Shao W., Lv L., Chai L., Qu L. (2022). Wnt/beta-catenin signaling confers ferroptosis resistance by targeting GPX4 in gastric cancer. Cell Death Differ..

[33] Cozza G., Rossetto M., Bosello-Travain V., Maiorino M., Roveri A., Toppo S., Zaccarin M., Zennaro L., Ursini F. (2017). Glutathione peroxidase 4-catalyzed reduction of lipid hydroperoxides in membranes: The polar head of membrane phospholipids binds the enzyme and addresses the fatty acid hydroperoxide group toward the redox center. Free Radic. Biol. Med..

[34] Yang W.S., SriRamaratnam R., Welsch M.E., Shimada K., Skouta R., Viswanathan V.S., Cheah J.H., Clemons P.A., Shamji A.F., Clish C.B. (2014). Regulation of ferroptotic cancer cell death by GPX4. Cell.

[35] Friedmann Angeli J.P., Schneider M., Proneth B., Tyurina Y.Y., Tyurin V.A., Hammond V.J., Herbach N., Aichler M., Walch A., Eggenhofer E. (2014). Inactivation of the ferroptosis regulator Gpx4 triggers acute renal failure in mice. Nat. Cell Biol..

[36] Song X., Wang X., Liu Z., Yu Z. (2020). Role of GPX4-Mediated Ferroptosis in the Sensitivity of Triple Negative Breast Cancer Cells to Gefitinib. Front. Oncol..

[37] Badgley M.A., Kremer D.M., Maurer H.C., DelGiorno K.E., Lee H.-J., Purohit V., Sagalovskiy I.R., Ma A., Kapilian J., Firl C.E.M. (2020). Cysteine depletion induces pancreatic tumor ferroptosis in mice. Science.

[38] Koppula P., Zhuang L., Gan B. (2021). Cystine transporter SLC7A11/xCT in cancer: Ferroptosis, nutrient dependency, and cancer therapy. Protein Cell.

[39] Wang Y., Zhao G., Condello S., Huang H., Cardenas H., Tanner E.J., Wei J., Ji Y., Li J., Tan Y. (2021). Frizzled-7 Identifies Platinum-Tolerant Ovarian Cancer Cells Susceptible to Ferroptosis. Cancer Res..

[40] Martinez A.M., Kim A., Yang W.S. (2020). Detection of Ferroptosis by BODIPY™ 581/591 C11. Methods Mol. Biol..

[41] Faustino-Rocha A., Oliveira P.A., Pinho-Oliveira J., Teixeira-Guedes C., Soares-Maia R., da Costa R.G., Colaço B., Pires M.J., Colaço J., Ferreira R. (2013). Estimation of rat mammary tumor volume using caliper and ultrasonography measurements. Lab Anim..

