# Supplementary material for: High-Dose Selenium Induces Ferroptotic Cell Death in Ovarian Cancer
Source: Int J Mol Sci. 2023 Jan 18;24(3):1918. doi: 10.3390/ijms24031918 (PMC9915545; doi:10.3390/ijms24031918)

Supplementary Figures

High-dose selenium induces ferroptotic cell death in ovarian cancer

Jung-A Choi, Elizabeth Hyeji Lee, Hanbyoul Cho, and Jae-Hoon Kim

Figure S1. Effect of high-dose SS on cell death in ovarian cancer cells

(A) SS-treated (24-h) SKOV3 cells stained with an Annexin V-FITC/PI for apoptosis. (B) SKOV3 cells were treated with single or repeated doses of SS (twice at 24-h intervals, halved SS dose of the single administration). Cell death was determined using an Annexin V-FITC/PI apoptosis detection kit.

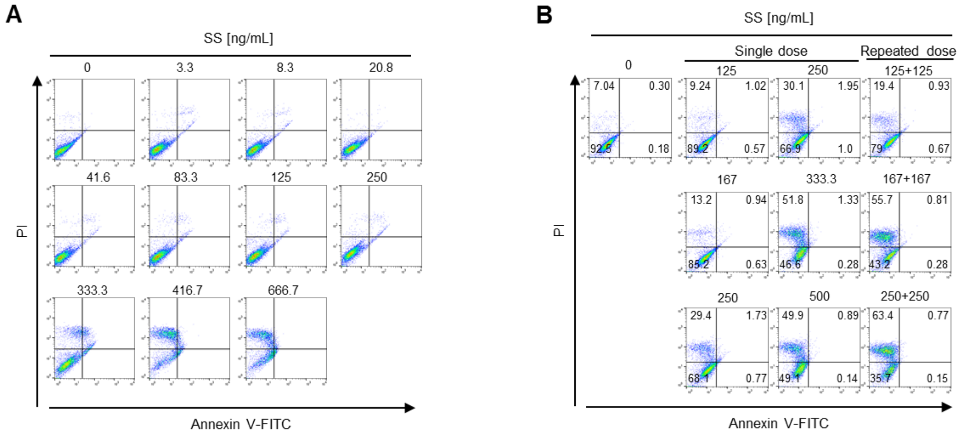

Figure S2. High-dose SS induces cytoplasmic vacuoles in ovarian cancer cells

SKOV3 cells were treated with single or repeated dose of SS. Repeated administration was performed twice at 24-h intervals with a halved dose as the single administration dose. Cells with differential morphologies were visualized using bright field microscopy.

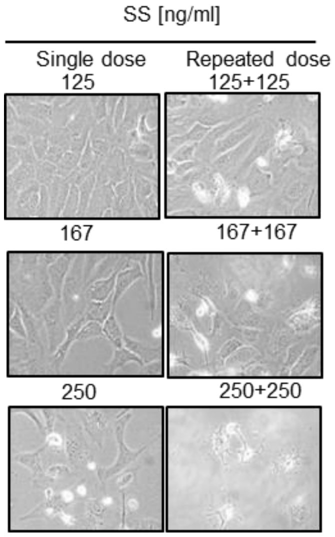

Figure S3. Effect of carboplatin and paclitaxel on morphological changes in SKOV3 cells

Cells were treated with either carboplatin (100 μg/ml) or paclitaxel (50 ng/ml). After 48 h, the cells were visualized using bright field microscopy

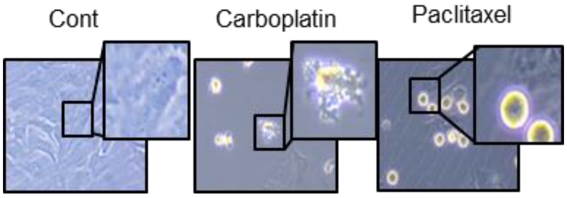

Figure S4. Effect of carboplatin on cell death in ovarian cancer cells

(A–C) SKOV3 cells were treated with carboplatin or paclitaxel for 24 or 48 h. Cell proliferation assay was performed by a Trypan Blue staining assay (A). Annexin V-FITC staining and PI incorporation were measured using a FACSCalibur flow cytometer (B) and analyzed using FlowJo software (C).

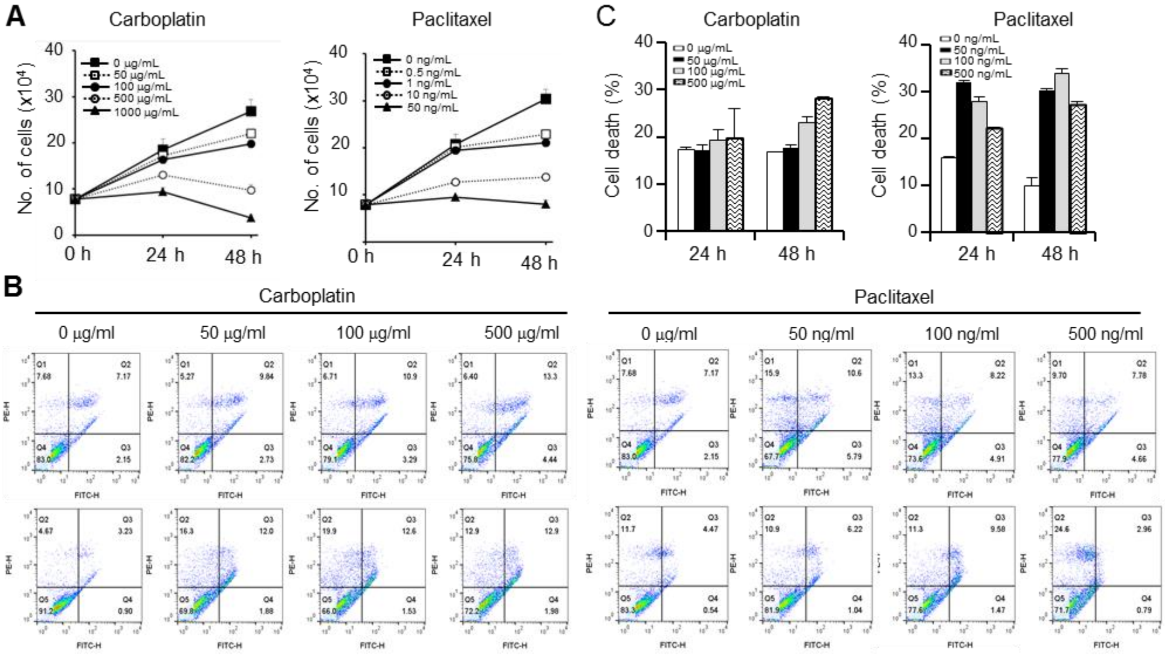

Figure S5. The impaired GPx expression by high-dose SS in SKOV3-bearing mice

Tumor tissue samples were harvested from mice and lysed for western blot analysis. Lysates were immunoblotted with GPx1 and GPx4 antibodies. GAPDH was used as the loading control. Quantification of immunoblots was analyzed by ImageJ programs. GAPDH was used as the endogenous control to normalize the data. Data are expressed as the means ± SD

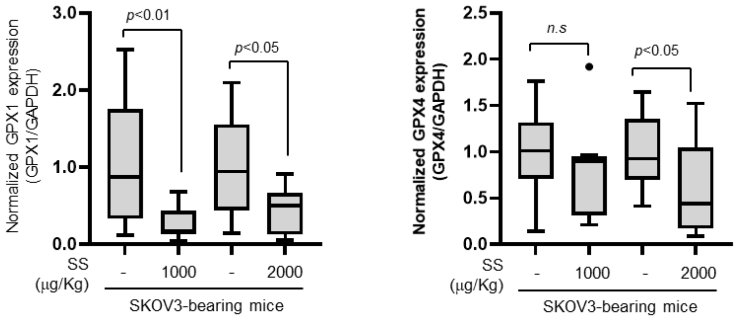

Figure S6. Effect of high-dose SS on cell death in IHOSE4138, IHOSE8695 and SKOV3 cells

(A–B) IHOSE4138, IHOSE8695 and SKOV3 cells were treated with SS 313 ng/mL in the presence or absence of ferrostatin-1 (50 μM) for 24. Annexin V-FITC staining and PI incorporation were measured using a FACSCalibur flow cytometer (A) and analyzed using FlowJo software (B).

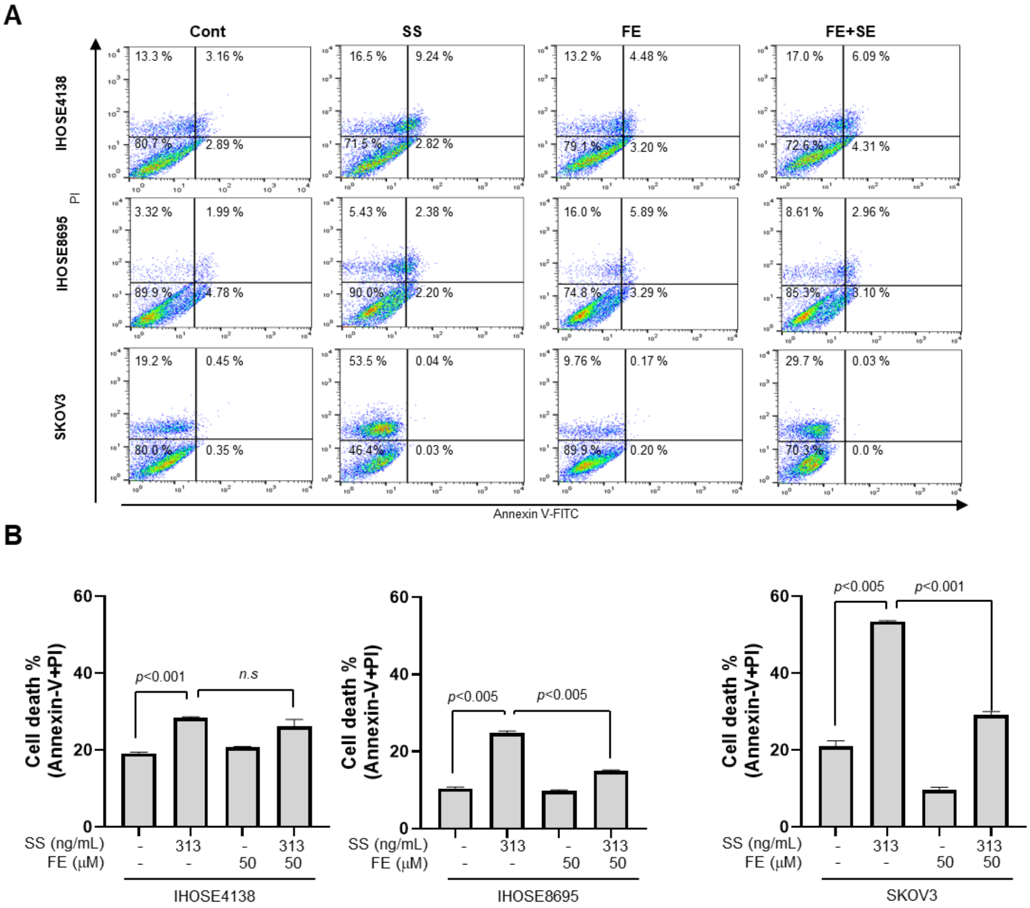

Supplement: Supplementary file 1 [file ijms-24-01918-s001.zip › ijms-2124643-supplementary.pdf]
